# Functional characterization of E- and P-cadherin in invasive breast cancer cells

**DOI:** 10.1186/1471-2407-9-74

**Published:** 2009-03-03

**Authors:** David Sarrió, José Palacios, Marta Hergueta-Redondo, Gonzalo Gómez-López, Amparo Cano, Gema Moreno-Bueno

**Affiliations:** 1 Department of Biochemistry UAM, Instituto de Investigaciones Biomédicas "Alberto Sols" (CSIC-UAM), C/Arturo Duperier 4. 28029, Madrid, Spain; 2 Breakthrough Breast Cancer Research Centre, The Institute of Cancer Research, 237 Fulham Road, SW3 6JB, London, UK; 3 Servicio de Anatomía Patológica, Hospital Virgen del Rocío, Avda, Manuel Siurot S/N, 41013, Sevilla, Spain; 4 Bioinformatics Unit, UBio, Spanish National Cancer Research Center, CNIO, C/Melchor Fernández Almagro, 28029 Madrid, Spain

## Abstract

**Background:**

Alterations in the cadherin-catenin adhesion complexes are involved in tumor initiation, progression and metastasis. However, the functional implication of distinct cadherin types in breast cancer biology is still poorly understood.

**Methods:**

To compare the functional role of E-cadherin and P-cadherin in invasive breast cancer, we stably transfected these molecules into the MDA-MB-231 cell line, and investigated their effects on motility, invasion and gene expression regulation.

**Results:**

Expression of either E- and P-cadherin significantly increased cell aggregation and induced a switch from fibroblastic to epithelial morphology. Although expression of these cadherins did not completely reverse the mesenchymal phenotype of MDA-MB-231 cells, both E- and P-cadherin decreased fibroblast-like migration and invasion through extracellular matrix in a similar way. Moreover, microarray gene expression analysis of MDA-MB-231 cells after expression of E- and P-cadherins revealed that these molecules can activate signaling pathways leading to significant changes in gene expression. Although the expression patterns induced by E- and P-cadherin showed more similarities than differences, 40 genes were differentially modified by the expression of either cadherin type.

**Conclusion:**

E- and P-cadherin have similar functional consequences on the phenotype and invasive behavior of MDA-MB-231 cells. Moreover, we demonstrate for the first time that these cadherins can induce both common and specific gene expression programs on invasive breast cancer cells. Importantly, these identified genes are potential targets for future studies on the functional consequences of altered cadherin expression in human breast cancer.

## Background

Cadherins are integral membrane glycoproteins that mediate calcium-dependent cell-cell adhesion [1]. Their genes cluster in specific chromosomal regions, such as human 16q22.1, where E-cadherin (*CDH1*) and P-cadherin (*CDH3*) genes are located [2]. Cadherins have important differences in tissue expression and function [3]. While E-cadherin (E-cadh) is expressed in the luminal epithelial cells of the normal breast, P-cadherin (P-cadh) expression is restricted to myoepithelial cells [3,4]. The cytoplasmic domain of the cadherins interacts with the catenins (α-, β-, γ-catenin and p120) to form the functional adhesion complexes [5]. The catenins, in turn, are able to mediate signaling pathways (such as the Wnt-pathway) that control the expression of a number of genes involved in tumorigenesis [6,7]. Alterations in the cadherin-catenin complexes are directly implicated in tumorigenesis and cancer progression [3,8,9]. E-cadherin gene (*CDH1*) is considered a tumor and invasion suppressor gene, since its down-regulation enhance cell migration and promotes metastasis in a variety of cancer models [10-13]. Reduction of E-cadherin expression is a frequent event in various carcinoma types [3,8,9]. In breast cancer, E-cadherin expression is reduced in about 50% of the ductal tumors, and completely lost in the majority of lobular carcinomas [14,15]. Loss of E-cadherin function in breast tumor cells can be achieved by genetic (loss of heterozygosity and inactivating mutations) and epigenetic (promoter hypermethylation) alterations [14,16]. Moreover, *CDH1 *can be transcriptionally repressed by a variety of transcription factors, such as Snail (Snai1), Slug (Snai2) and Twist, among others (reviewed in [17]). In some tumor types and cellular models, loss of E-cadherin associates to the process known as Epithelial-Mesenchymal Transition (EMT), which is characterized by up-regulation of mesenchymal markers (vimentin, fibronectin), modification of actin cytoeskelton (induction of stress fibers and focal adhesions), and acquisition of a fibroblastic, highly invasive and metastatic phenotype [17,18].

Contrary to E-cadherin, P-cadherin positive expression in breast carcinomas associates to unfavorable prognostic factors, such as high grade and estrogen receptor negativity [19-22], but the functional role of P-cadherin in breast tumorigenesis is poorly understood. Moreover, the exact mechanisms by which the cadherins control tumor invasion are not well defined.

To gain insights into the functional role of the E- and P-cadherin in invasive breast cancer, we have analyzed the effects of the ectopic expression of these cadherins on the phenotype, migratory and invasive behavior of the MDA-MB-231 cell line (hereafter referred as 231). Moreover, we have characterized for the first time the transcriptional changes modulated by E- and P-cadherin in these cells, thus providing novel potential targets secondary to altered cadherin expression in invasive breast cancer.

## Methods

### Cell Culture and Transfection

MDA-MB-231 and MCF7 breast cancer cell lines were obtained from the American Type Culture Collection (ATCC), and grown in DMEM medium, supplemented with 10% fetal calf serum, and 1% penicillin-streptomycin. To generate stable cell lines, cells were transfected with the Dexamethasone (Dex)-inducible pLK-pac-based vectors containing the complete human E-cadherin or P-cadherin mRNAs [23] as well as the empty vector, using TransFast Reagent (Promega). The plasmids were kindly provided by Dr Albert B. Reynolds, (Nashville, USA), and Keith R. Johnson (Omaha, USA). By puromycin selection we isolated two clones for each condition: stably transfected with E-cadherin (hereafter referred to as 231-E-cadh), with P-cadherin (231-P-cadh) or containing the empty vector (231-Control). To induce cadherin expression, cells were treated with 10 nM Dexamethasone for 24 hours. All experiments were performed using two clones for each condition, and the presented data represents the mean value of them.

### Immunofluorescence

Cells grown on coverslips were fixed in either methanol (-20°C) or 3.7% formaldehyde (30 min at room temperature) and then incubated with the primary antibodies at the appropriate dilutions. The primary antibodies used were: mouse anti-E-cadherin (clones HECD1 and 4A2C7, from Zymed), anti-P-cadherin (BD Biosciences), anti β-catenin (BD Biosciences), anti-paxillin (Calbiochem) anti-vimentin (DAKO), anti-cytokeratins (AE1/AE3 from Biomeda), and polyclonal rabbit anti-p120 (Sta Cruz Biotechnology). The Alexa-488-coupled phalloidin (Molecular Probes, Eugene, OR) was used to stain actin cytoskeleton. Secondary antibodies were Alexa488-coupled anti-mouse, Alexa594-coupled anti-mouse or FITC-coupled anti-rabbit (Molecular Probes). Cell nuclei were stained using 4,6-diaminidino-2-phenylindole (DAPI) (Molecular Probes). Staining was examined using a confocal ultra-spectral-microscope (TCS-SP-2-AOBS-UV, Leica, Wetzlar, Germany). Post-capture image analysis and processing of confocal image stacks were performed using the Leica Confocal software.

### Western Blot

For western blotting, cells were washed with HMF Buffer (*Hepes buffer Mg^2+ ^Free*, 10 mM Hepes pH 7.4, 150 mM NaCl, 1 mM CaCl_2_, 0.1% glucose) and proteins were extracted using standard RIPA buffer containing protease inhibitors. Polypeptides were transferred onto Immobilon-P membranes (Millipore, Bedford, MA), and blots were then incubated with anti-E-cadh (1:1000), anti-P-cadh (1:500) or alfa-tubulin (1:10,000) monoclonal antibodies for 1 hour. After washing, anti-mouse Ig HRP-conjugated secondary antibody (Amersham, Buckinghamshire, UK) was then applied, and visualized using the ECL chemiluminescence detection kit (Amersham Biosciences, Uppsala, Sweden). For detection of E-cadherin and P-cadherin proteins, blots were exposed 5 minutes, for alfa-tubulin 30 seconds. To compare the levels of exogenous cadherins in 231-E-cadh and P-cadh clones, band intensity was measured using ImageJ software. Relative to alfa-tubulin (loading control), cadherin expression after Dex induction was 0,98 and 0,92 for 231_E-cadherin clones, and 1,0 and 1,1 for P-cadherin clones.

### RNA extraction, amplification, labelling and microarray hybridization

Control cells (2 clones), as well as E-cadherin (2 clones) and P-cadherin (2 clones) transfectans were treated with Dex for 48 h and then grown without Dex for another 24 h, until the cells reached complete confluence. Moreover, control cells were also grown in parallel for 72 h without Dex (control untreated), until reached confluence. Total RNAs for each condition were extracted using RNAesy Extraction Kit (QIAGen), and subsequently amplified by *in vitro *transcription using Megascript T7 protocol (Ambion, Austin, TX), essentially as reported before [24,25]. Amplified RNAs for the three control clones treated with Dex were then mixed in equal proportions to obtain a "Control-treated pool". This pool was labeled with Cy3-dUTP fluorochrome (Amersham, Uppsala, Sweden) and subsequently used as the "reference sample" in the hybridizations. Hybridizations were performed onto the "CNIO Oncochip" cDNA microarray v 2.0, as described previously [24,25].

Since the addition of Dex alone may alter the transcriptional level of some genes, to identify the genes whose expression was directly modified by E-cadherin or P-cadherin induction, we first hybridized (in quadruplicate) Cy3-labelled-Control-treated pool against Cy5-Control-untreated cells. The genes modulated by at least 2-fold after Dex treatment in 231 control cells (data not shown) were removed from the following analysis. After this filtering, the amplified RNA from each of the 231_E-cadh and 231_P-cadh clones was labeled with Cy5 and then hybridized against a pooled RNA from the three Dex-treated control clones (reference sample). Two independent hybridizations were performed for each clone, thus a total of four experiments were performed for each condition (E-cadh or P-cadh transfected cells). Microarray slides were washed, dried, and then scanned in a Scanarray 5000 XL scanner (GSI Lumonics, Kanata, Ontario, Canada).

### cDNA microarray data analysis

Data from the fluorescence intensity measurements of each array experiment were processed using GenePix Pro 5.0 program (Axon Instruments, Inc., Union City, CA). For statistical analysis, we selected genes with expressions that differed by a factor of at least 2-fold with respect to the control reference. A hierarchical clustering method was applied to group of genes and samples on the basis of the similarities in expression, and the unsupervised analysis were visualized using the SOTA and TreeView software http://gepas3.bioinfo.cipf.es/cgi-bin/sotarray, http://gepas3.bioinfo.cipf.es/cgi-bin/treeview, assuming Euclidean distances between genes. Genes with potentially significant changes in expression among the conditions were identified using the POMELO program http://pomelo2.bioinfo.cnio.es/. We tested the null hypothesis of equal means among the three groups using ANOVA, computing p-values using a permutation test. To select differentially expressed genes, we adjusted for multiple testing using the False Discovery Rate (FDR) method; we report as differentially expressed genes with FDR adjusted p-value < 0.15.

### Protein interaction network

Modified genes found in the microarrays data were used to produce a Protein-Protein Interaction (PPI) network for the biological processes level. This analysis was carried out using PathArchitect software v4.0 (Stratagene) incorporating information about these genes. Relevance networks were built by using the algorithm indicated by the manufacturer.

### Real time quantitative RT-PCR (qRT-PCR)

To validate data from cDNA microarrays, the expression of selected genes was analyzed by qRT-PCR using gene-specific TaqMan probes (Applied Biosystems, Foster City, CA). Analysis was performed with the ABI PRISM 7700 Sequence Detection System Instrument and software (Applied Biosystems), following the manufacturer's recommendations. Each reaction was performed in triplicate from two cDNA dilutions. The internal standard human beta-2 microglobulin gene (*B2M*; Applied Biosystems) was used to normalize cDNA quantity. The amount of target gene and endogenous reference was determined using the standard curve method. The standard curve was constructed by five-fold serial dilution of cDNA from a pool of breast cancer tissues.

### Cell aggregation Assay

MCF7 and 231 cells were tested for their ability to aggregate in hanging drop suspension cultures, essentially as described previously [23]. Briefly, cells were trypsinized, counted, resuspended in DMEM medium with or without 10 nM Dex and plated in equal numbers as hanging drop cultures. Cells were allowed to aggregate for 24 h at 37°C, and were then pipetted 10 times to disrupt weak aggregates prior to photography. The size of the particles was measured using ImageJ 1.37c software and the mean particle area was represented for each of the experimental conditions (n = 25).

### In vitro wound healing assay

Equal number of cells was plated in triplicate on 60 mm dishes. Cells were grown in culture media containing 10 nM Dex for 48 hours, until the cells reached the confluence. Monolayers of confluent cultures were lightly scratched with a 20 μl-pipette tip and were photographed by phase-contact microscopy at timed intervals for up to 24 hours. Quantification was done by measuring the number of pixels in the wound area using Adobe^® ^Photoshop^® ^and calculating the decrease in the area by subtracting the number of pixels from the number of pixels in the corresponding wound area at the 0 hour time point. Additionally, wound healing assays were filmed by videomicroscopy, using a confocal microscope (TCS-SP-2-AOBS-UV, Leica). Videos were assembled using Adobe^® ^Premiere Pro 1.5 software.

### Matrigel Transwell invasion assays

Cells were grown with 10 nM Dex during 48 h. To minimize degradation of the cadherins, cells were shed using EDTA-free Trypsin solution (Sigma), supplemented with 0.5 mM CaCl_2_. Cells were suspended in 500 μl DMEM medium containing 0.1% FBS and 10 nM Dex. The ability to invade through extracellular matrix was assessed by Matrigel-coated Transwell invasion chambers of 8 μm pore size (BD BioCoat Matrigel Invasion Chamber, Becton Dickinson) following manufacturer's instructions. 5 x 10^5 ^cells were plated on the Transwell, and the lower plate contained DMEM 10% FBS as chemoattractant. After 24 h cells in the upper filter were removed and the cells that had traversed to the lower surface of the filter were fixed and stained with DAPI. The number of migrated cells per x20 field was analyzed microscopically and counted with Scion Image Software (Scion Corporation). Experiments were performed in duplicate, using two clones for each condition.

## Results

**Figure 1:**
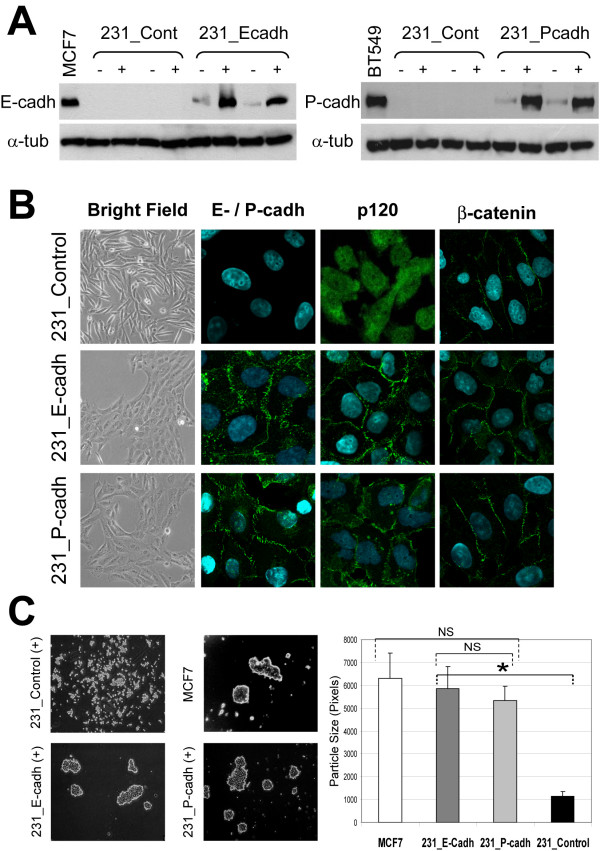
**Exogenous expression of E- and P-cadherin promotes formation of functional *adherens junctions *and morphological changes in 231 cells**. **A**: Western blot showing E- and P-cadherin expression before (-) and after (+) addition of Dexamethasone in 231 clones. MCF7 and BT549 cells were used as controls for endogenous expression of E- and P-cadherin, respectively. **B: **E- or P-cadherin expression promotes epitheloid morphology (first column, bright field) and recruits the catenins (p120 and beta-catenin) to the *adherens junctions *(immunofluorescence, columns two to four). **C: **E- or P-cadherin expression enhances cell aggregation. Left pictures show representative examples of cellular aggregation in each condition ("-", before and "+" after addition of Dex). E-cadherin positive MCF7 cells were used as control. Right: Quantification of mean particle size. Each bar represents mean ± SEM size (measured in pixels) of cell aggregates (n = 25, for each condition). Mean differences were compared by Student's t test (* indicates differences statistically significant p < 0.05; NS, not significant).

To assess the functional role of E-cadherin and P-cadherin in invasive breast cancer, we first transfected 231 cells with a constitutively activated expression vector (pCDNA3) containing either the complete human E-cadherin or P-cadherin cDNAs (kindly provided by Dr Setsuo Hirohashi, Tokyo, Japan) [26]. However, we failed to obtain stable clones expressing exogenous cadherins in the cell membrane, and most of the cadherin protein remained within the Golgi network (not shown). Moreover, because high levels of E-cadherin are sometimes inhibitory to cell growth [27,28], we then decided to use a Dexamethasone (Dex)-inducible expression system (Plk-pac, kindly provided by Drs A. Reynolds, (Nashvile, USA), and Keith R. Johnson (Omaha, USA)). This expression system has been previously demonstrated to induce dose-dependent cadherin expression in 231 cells and other cadherin-deficient cell lines [23]. After transfection, isolated 231-E-cadh and 231-P-cadh clones expressed low (basal) levels of E-cadh or P-cadh, respectively, as determined by Western blot (Figure 1A). In these cells, Dex treatment increased the expression of the cadherins around 10-fold, but not detectable expression was observed in control cells (Figure 1A). Measurement of band intensity demonstrated similar expression of E-cadherin and P-cadherin in all the clones used (see Materials and Methods).

Moreover, induction of E-cadherin or P-cadherin expression promoted a remarkable change in the morphology of 231 cells, which formed epithelium-like clusters, whereas control clones maintained the fibroblastic appearance (Figure 1B). By immunofluorescence, exogenous E-cadh and P-cadh were mainly localized at the cell-to cell contacts. Moreover, whereas in control cells beta-catenin was expressed at low levels in the cell membrane and p120 accumulated in cytoplasm and nucleus [23], in cadherin-expressing cells β-catenin was strongly expressed at cell-cell contacts, and p120 shifted from the cytoplasm to the membrane, where it co-localized with P-cadh or E-cadh (Figure 1B). To demonstrate the functionality of these *adherens junctions*, we performed a classic cell aggregation assay. After 24 h of Dex induction, E- and P-cadh-expressing clones showed prominent aggregates, considerably bigger than control clones, but comparable to those formed by the E-cadherin-positive breast cancer cell line MCF7 (Figure 1C). 231-E-cadh cells aggregates were slightly bigger than those formed by 231-P-cadh cells, but the differences were not statistically significant (Figure 1C).

**Figure 2:**
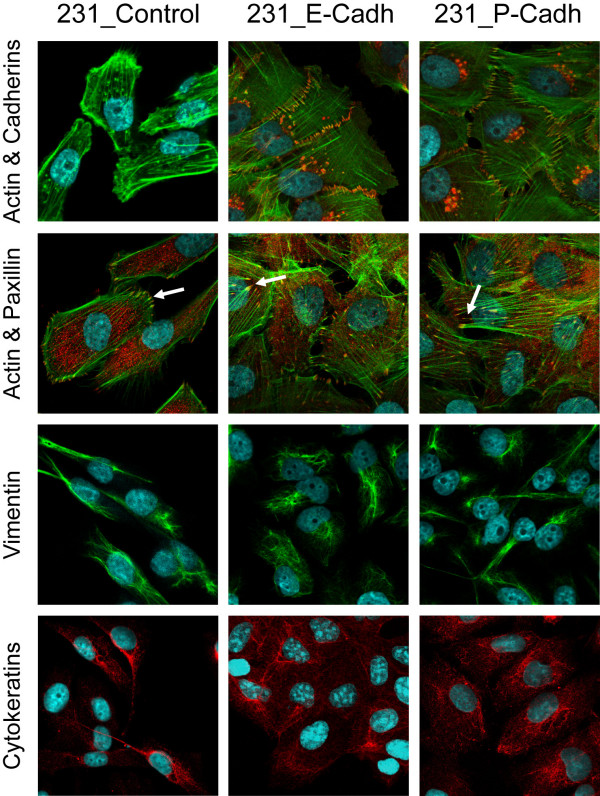
**Expression of E- or P-cadherin does not completely reverse the mesenchymal phenotype of 231 cells**. First row: Double immunofluorescence of E- or P-cadherin (Alexa-594, red) and actin cytoskeleton (Phalloidin-Alexa-488, green). Second row: Focal adhesions (arrows) co-stained with Phalloidin (green) and anti-paxillin (red). Note that no evident changes in the number or organization of focal adhesions are seen among the different conditions. Third and fourth rows: expression of the mesenchymal marker vimentin and the epithelial marker cytokeratin.

We next studied whether expression of these cadherins could completely reverse the fibroblastic phenotype of 231 cells. Expression of these cadherins did not significantly modify actin cytoskeleton organization or the number of focal adhesions (Figure 2). Moreover, no evident changes in the expression of EMT markers, such as cytokeratins or vimentin were found (Figure 2), thus indicating that E- and P-cadh expression is not sufficient to induce a complete epithelial phenotype in these cells.

**Figure 3:**
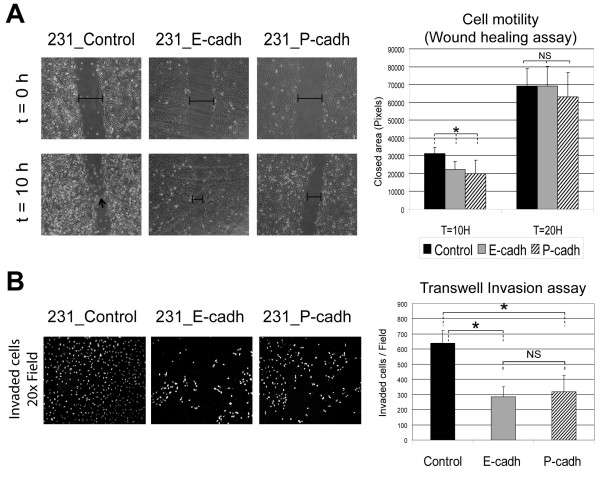
**E- and P-cadherin modify the migratory and invasive behaviour of 231 cells**. **A: **Study of cell migration by wound healing assay. Left pictures show that control cells migrate as single cells (arrow) whereas 231_E-cadh and 231_P-cadh cells migrate as clusters. Right: Cell migration was quantified by measuring the wounded area (in pixels) that was covered by the cells in the indicated time points. Each bar represents mean ± SEM of four independent experiments. **B: **Invasion ability through extracellular matrix (Matrigel) assessed by Transwell assays. Left: Representative images showing the number of invaded cells (nuclei stained by DAPI) after 24 hours. Right: Quantification of invasion. Each bar represents mean number ± SEM of invaded cells for each condition (four independent experiments). Mean differences were compared by Student's t test (* indicates differences statistically significant p < 0.05; NS, not significant).

We then evaluated whether expression of these cadherins altered cell motility using a classical wound-healing assay. At early time points (10 h) we observed a significant decrease in the migration ability of E-cadh and P-cadh-positive cells compared to control cells (Figure 3A). However, both 231-E-cadh and P-cadh cells finally closed the gap at similar time point as the control cells did (Figure 3A). No statistical differences between E-cadh and P-cadh clones were observed. Interestingly, using videomicroscopy, we observed that control cells infiltrate the wounded area as single cells (Figure 3A, marker by arrow; and see Additional file 1), whereas E- or P-cadh cells moved together as clusters, in a collective fashion (see Additional files 2 and 3). Therefore, expression of E-cadh or P-cadh alters the pattern of cell migration of 231 cells by reducing their ability to infiltrate as single cells and promoting an epithelial cell sheet-like movement.

Additionally, to assess the invasive capacities of cadherin-transfected cells we performed *in vitro *invasion assays using Matrigel-coated Transwell chambers. As shown in Figure 3B, 231-E-cadh as well as 231-P-cadh cells invaded through the extracellular matrix considerably less than 231-Control cells. No significant differences were observed between 231-E-cadh and 231-P-cadh cells, thus indicating that in the 231 cellular model both cadherins show similar invasion suppressor abilities.

Since these cadherins were able to modify cellular morphology, the migratory pattern, and invasive potential, it raised the question of whether these molecules could mediate their effects, at least in part, through regulation of gene expression at the transcriptional level. To study if the establishment of E-cadherin and P-cadherin-dependent cell adhesion leads to regulation of gene expression, we performed cDNA microarrays experiments (see detailed procedures in Materials and Methods).

Using this approach, 126 clones (representing 107 known genes) were found to be up- or down-regulated at least 2 fold in 231-E-cadh cells with respect to control cells, whereas in 231-P-cadh cells 98 clones (79 genes) were modulated (Table 1). According to data bases (Gene Cards; http://www.genecards.org) these genes belonged to a wide a range of biological functions, including signal transduction and growth factors (*VEGFC*, *FGFR4*), cell cycle (*CCNA2*), cell adhesion and extracellular matrix (*CDH4*, *COL12A1*), or cytokines and inflammation (*IL24*), among others. This indicates that in addition to their role in cell-adhesion, E-cadh and P-cadh have a significant impact on the overall genetic program of breast cancer cells. Table 2 shows a selection of genes, the complete list of genes, arranged by function is provided in Additional file 4.

**Table 1:** Functional grouping of genes modulated at least 2 fold in E-cadherin or P-cadherin-expressing cells with respect to control cells.

	231-E-cadherin			231-P-cadherin		
**Gene function**	**Up**	**Down**	**All**	**Up**	**Down**	**All**

Basic cellular functions	15	8	23	13	8	21

Cell cycle and Apoptosis	3	14	17	2	4	6

Signal Transduction	6	14	20	9	6	15

Adhesion & ECM	1	8	9	2	3	5

Cytokines & Immune	1	7	8	2	1	3

Metabolism	7	2	9	4	2	6

Transcription related	0	6	6	4	4	8

Motility & Cytoskeleton	4	3	7	4	1	5

Unknown function	3	5	8	2	8	10

TOTAL	**40**	**67**	**107**	**42**	**37**	**79**

**Table 2:** Selection of genes modulated at least 2 fold after E-cadherin and/or P-cadherin induction with respect to control cells.

GenBank accession #	Gene Symbol	Description	Mean E-cadh	Mean P-cadh
**Cellular adhesion and Extracellular Matrix**				
AA865745	***CDH18***	Cadherin 18, type 2	2.07	1.09
AI003692	***CDH4***	Cadherin 4, R-cadherin (retinal)	2.03	-1.20
H11732	*CLECSF2*	C-type lectin domain 2, member B	4.45	3.86
AA478481	*COL12A1*	Collagen, type XII, alpha 1	2.90	2.01
AW467461	*ITGB2*	Integrin beta 2	-2.28	-1.98
N33214	***MMP14***	Matrix metalloproteinase 14	-1.24	-2.46
N59721	***SERPINE2***	Plasminogen activator inhibitor type 1 member 2	-1.38	-2.87
**Motility and cytoskeleton**				
CR936693	*ADD1*	Adducin 1 (alpha)	-3.93	-4.66
N49912	*ARHGDIB*	Rho GDP dissociation inhibitor beta	-2.41	-6.07
AA009697	*CDC42*	Cell division cycle 42	2.03	1.73
AA148200	*ILK*	Integrin-linked kinase-2	2.09	1.66
**Signal transduction**				
AI140863	***EDN2***	Endothelin 2	3.12	1.10
AA446994	***FGFR4***	Fibroblast growth factor receptor 4	-1.17	-2.06
N20338	*HGS*	Hepatocyte growth factor-regulated tyrosine kinase substrate	2.16	1.53
AA026831	***KDR***	Kinase insert domain receptor	3.33	1.09
W55872	*NFKBIA*	NFKB inhibitor, alpha	2.25	1.66
H26426	***PTPRM***	PTP, receptor M	1.23	-2.05
H07991	*VEGFC*	Vascular endothelial growth factor C	2.20	2.61
**Transcription related**				
H21041	*ATF3*	Activating transcription factor 3	2.31	1.65
AA026120	***BHLHB2***	Basic helix-loop-helix domain containing class B2	1.04	-2.11
AA868008	*HIST1H4C*	Histone 1, H4c	2.50	2.15
**Cell Cycle and apoptosis**				
AA489752	***CCNG2***	Cyclin G2	2.18	-1.73
AA486628	*EGR1*	Early growth response 1	4.20	1.53
N94468	***JUNB***	Homo sapiens jun B proto-oncogene	2.05	1.04
AA826373	*RRM2*	Ribonucleotide reductase M2	3.30	5.53
AA039640	*WEE1*	WEE1 homolog	4.78	2.24
**Cytokines and immune response**				
W46900	*CXCL1*	Chemokine (C-X-C motif) ligand 1	3.56	2.58
N31850	***IL24***	Interleukin 24	2.88	-2.16

Genes are grouped by function and arranged by alphabetic order. Genes differentially expressed with statistically significance (FDR < 0.15) between E-cadherin and P-cadherin are highlighted in bold letter. Mean E-cadh: mean gene expression value in two E-cadherin clones; Mean P-cadh: mean gene expression value in two P-cadherin clones. Negative value indicates that gene expression is down-regulated with respect to control cells.

Moreover, to identify possible signaling pathways regulated by E- and P-cadherin, we have generated potential protein-protein interaction networks with cadherin-regulated genes at the biological processes level (Additional files 5 and 6). This analysis demonstrated that some cadherin-regulated genes are interconnected, thus providing numerous cross-talks among distinct biological processes.

**Figure 4:**
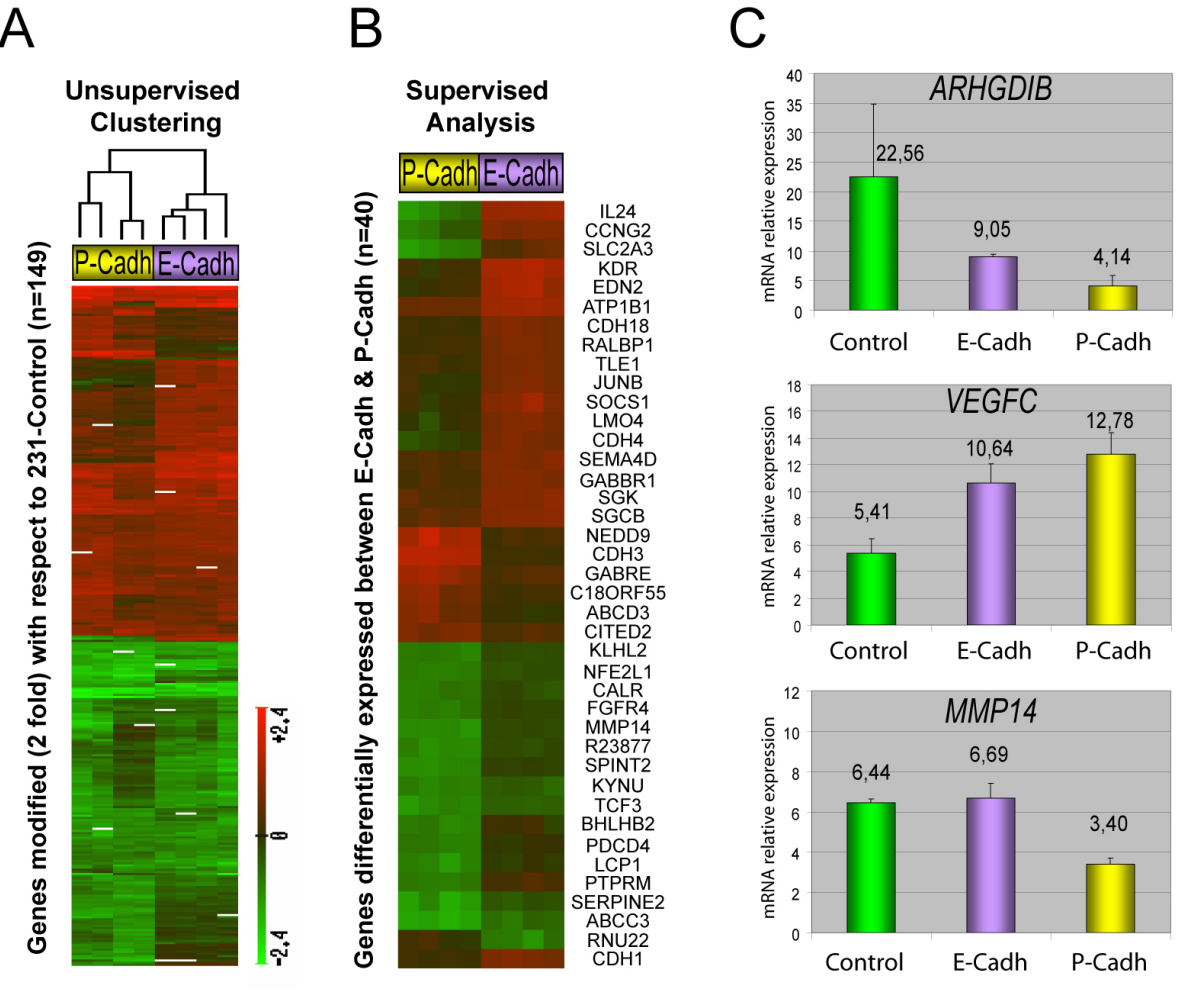
**E- and P-cadherin modulate transcriptional changes**. **A**: Unsupervised hierarchical clustering of the gene expression patterns induced by E- or P-cadherin expression. Columns represent microarray experiments, and rows gene expression of the 149 genes modified at least two-fold with respect to control cells. Intensity of color is a function of the gene expression level as depicted in the scale bar. **B: **Identification of genes differentially expressed (with a statistical significance of FDR < 0.15) between 231_E-cadh and 231_P-cadh cells. **C: **Validation of microarray data of selected genes by qRT-PCR. Bars represent mean gene expression ± SEM (mRNA levels relative to control *B2M *transcript) from four different experiments.*ARHGDIB *is down-regulated and *VEGFC *is over-expressed in 231_E-Cadh and 231_P-cadh cells compared to control cells. *MMP14 *is down-regulated only in 231_P-cadh clones.

Then, to visually compare the expression profiles modulated by each cadherin type we performed an unsupervised hierarchical clustering. As shown in Figure 4A, the four hybridizations of 231-E-cadh cells grouped together in a branch separated from that of 231-P-cadh cells, suggesting that differences in their expression profiles may exist. However, using this clustering method more similarities than differences were observed between the expression profiles of 231-E-cadh and 231-P-cadh cells. To better establish which genes were differentially and specifically regulated by each cadherin type, we performed a supervised analysis using the POMELO tool. Using this ANOVA-based supervised approach, 46 clones (40 genes) were identified as differentially expressed with a significant association (FDR-adjusted p value < 0.15) between the two cadherin types (Figure 4B; complete list of genes in Additional file 4). Therefore, these data indicate that E- and P-cadherin induce a common gene expression program, but they can also specifically modify a subset of genes.

Finally, real-time quantitative RT-PCR analysis (qRT-PCR) on selected genes validated the microarrays data (Figure 4C). Specifically, we confirmed the down-regulation of the Rho GDP dissociation inhibitor beta gene (*ARHGDIB*) in both 231-E-cadh and 231-P-cadh cells (although to a lesser extent in 231-E-cadh cells), and the over-expression of Vascular endothelial growth factor C (*VEGFC*) in cells expressing either cadherin type. Moreover qRT-PCR analysis also confirmed the differential regulation of Matrix Metalloproteinase 14 gene (*MMP14*); down-regulated in 231-P-cadh but not in 231-E-cadh cells.

## Discussion

Abnormal expression of diverse cadherin types has been widely reported in breast cancer [3,8,9], but the causes and functional consequences of these alterations are not fully understood. Our data indicate that exogenous expression of either E- or P-cadherin in highly invasive MDA-MB-231 cells promotes a switch from fibroblastic to epitheloid morphology. However, although these cadherins are unable to completely reverse the mesenchymal phenotype in invasive breast cancer cells (as evidenced by the maintenance of multiple actin stress fibers, focal adhesions, and the expression of vimentin [[29,30] and current work]), we show here that either E- or P-cadherin reduces the ability of 231 cells to infiltrate as single cells, by promoting an epithelial-like collective cell migration. Moreover, we confirmed the invasion suppressor activity of E-cadherin [30-32], and demonstrate that P-cadherin can also reduce invasion in breast cancer cells, in the same way as reported in lung cancer [33] and melanoma cells [34]. However, the anti-invasive potential of E- or P-cadherin in cancer cells may vary among the different cancer cell types and biological contexts. For instance, a high level of exogenous P-cadherin over-expression, or the presence of endogenous classical cadherins might be responsible, in part, for the pro-invasive or pro-migratory effect of P-cadherin observed in epithelial breast MCF7/AZ or pancreatic Panc-1 cells [35,36]. It should be recalled that the different types of cadherins compete for a limited pool of catenins (especially p120), thus provoking cadherin endocytosis [37]. To minimize this competitive effect we have used the 231 cell line, which lacks classical cadherins [38], and an inducible system that allowed expressing the exogenous E- or P-cadherin to levels similar to the endogenous expression in other breast cancer cell lines.

Assuming the particularities of the different cellular models, increasing evidences indicate that the roles of the cadherins in carcinogenesis and tumor progression, do not solely lie on their adhesive function [30-32,39], but also depend on their interaction with other molecules (such as cytoskeletal components, integrins, and growth-factor receptors, among others) and signaling pathways [6,7,40,41]. Therefore, it has been proposed that the cadherins may participate in a variety of signaling pathways leading to modulation of specific genes at the transcriptional level, and as a consequence, regulation of important cellular functions, including invasion, motility, or cell cycle [8,13].

To demonstrate that the establishment of E-cadherin and P-cadherin-dependent cell adhesion leads to regulation of gene expression we performed a comprehensive gene expression analysis using large-scale cDNA microarrays. We detected the transcriptional modification (at least 2-fold) of 107 and 79 genes by E-cadherin and P-cadherin, respectively. The genes belonged to a variety of biological functions such as cell motility and invasion (*Cdc42*, *ITGB2*), cell cycle (*EGR1*, *RRM2*) or signal transduction (*VEGFC*), indicating that expression of E-cadh and P-cadh in breast cancer cells have a significant impact on their overall genetic program. Importantly, some of these cadherin-regulated genes may be functionally interconnected (Additional files 5 and 6) thus providing potential signaling pathways involved in distinct biological processes.

It is worth noting that a substantial number of genes were modified in a similar way by both cadherin types, suggesting the existence of a core "cadherin-associated" genetic program that would support the analogous functional consequences of expressing of E- or P-cadherin in 231 cells. Nonetheless, gene expression profiling analyses also demonstrated that each cadherin type can also specifically modify a subset of genes, thus indicating that they can regulate both common and specific biological functions in invasive breast carcinomas.

Focusing on cell motility and invasion, we observed that in 231 cells E- and/or P-cadherin regulate the expression of some genes associated to integrin signaling (*ITGB2*, β2 integrin; *ILK*, Integrin-linked kinase), intercellular adhesion (cadherins 4 and 18), extracellular remodeling (*MMP14*, *SERPINE2*), and cytoskeleton (*Cdc42*, *ARHGDIB*). Similarly, E-cadherin has been reported to transcriptionally regulate a variety of integrins and matrix metalloproteases (*MMP1*, *MMP2*, *MMP3*, *MMP14 *and *TIMP-1*) in other cancer models [42-45]. This indicates the existence of a functional crosstalk between cadherin, integrin signaling, and extracellular matrix remodeling in human tumors. This crosstalk, which can be controlled at the transcriptional level by the cadherins, may underlie the anti-invasive and anti-metastatic effect of the E-cadherin (and probably P-cadherin) in a variety of carcinomas. Consistently, E-cadherin loss increases the expression of α2, α3 and β1-integrins, MMP2 and MMP9 metalloproteases, and subsequently leads to an enhanced invasion and metastatic ability in transformed keratinocytes [45,46]. The exact mechanisms and signaling pathways leading to regulation of invasion by the cadherins are still to be clarified. Here we identify *ARHGDIB *(Rho GDP dissociation inhibitor beta, also known as D4-GDI) gene as a candidate gene potentially involved in this process. This gene, which encodes for a regulator of actin cytoskeleton, is transcriptionally down-regulated after E- and P-cadherin induction in 231 cells. Importantly, previous reports have demonstrated that inhibition of *ARHGDIB *in invasive breast cancer cell lines significantly reduces their invasive behavior through regulation of β1-integrin signaling [47].

Finally, it is important to highlight that the effect of the cadherins on the overall gene expression program of cancer cells is highly dependent on the cellular type and the biological context. Thus, cadherin regulation of specific transcriptional factors may depend on the activation of other signaling pathways, or the presence of additional molecular alterations. For instance, exogenous E-cadherin expression considerably alters the global gene expression profile of colon cancer cells with activated Wnt pathway, mainly by controlling the nucleo-cytoplasmic pool of beta-catenin. However, E-cadherin expression has a modest transcriptional effect on human fibroblasts [48]. Moreover, as mentioned before, E-cadherin alone is not able to completely reverse the mesenchymal phenotype of invasive breast cancer cells, or the full EMT induced by Snail in MDCK cells [49]. By contrast, in immortalized human mammary epithelial cells, shRNA-mediated E-cadherin knockdown resulted in complete EMT through the modulation of a large set of genes, whereas introduction of a truncated dominant-negative E-cadherin construct failed to do so [39]. Interestingly, in this cellular model, the induction of EMT after E-cadherin depletion seemed to be partially mediated by *Twist *and *ZEB1*, two strong E-cadherin repressors. Additionally, in the MDA-MB-435 breast cancer cell line the presence of wild-type or mutant E-cadherin proteins leads to the differential regulation of a variety of EMT inducers (*Snail*, *Twist*, *ZEB1*, *TCF3*) [44]. We observed here that *TCF3 *mRNA is down-regulated in 231 cells expressing P-cadherin but not E-cadherin. Together all these data suggest that the levels of different cadherin types or the presence of abnormal (mutant) cadherins might lead to the differential regulation of EMT transcription factors in breast cancer cells. These factors, in turn, might control cadherin transcriptional levels, thereby generating an auto-regulatory loop. However, this hypothesis has not been tested yet.

## Conclusion

Our data indicate that in fibroblastic invasive breast cancer cells, both E- and P-cadherin have similar effects on the cell phenotype and the migratory and invasive behavior. Moreover, the establishment of E- and P-cadherin-dependent cell adhesion leads to regulation of a common gene expression program, and modulation of particular genes that may potentially underlie their anti-invasive effect. In addition, they can also specifically modify a subset of genes, thus suggesting that they could mediate both common and specific biological functions in invasive breast carcinomas. The identified genes are potential targets for future studies on the functional consequences of altered cadherin expression in human breast cancer.

## Abbreviations

E-cadh: E-cadherin; P-cadh: P-cadherin; Dex: Dexamethasone.

## Competing interests

The authors declare that they have no competing interests.

## Authors' contributions

DS carried out molecular, cell biology, and microarray experiments, participated in the design of the study and drafted the manuscript. JP participated in the design of the study and drafted the manuscript. MH-R participated in microarray experiments and data analysis. GG participated in the protein interaction networks. AC participated in the study design and drafted the manuscript. GM-B conceived the study, performed microarray data analysis, and drafted the manuscript. All authors read and approved the final manuscript.

## Pre-publication history

The pre-publication history for this paper can be accessed here:

http://www.biomedcentral.com/1471-2407/9/74/prepub

## Supplementary Material

Additional file 1 **Wound healing assay 231-control cells**. Wound healing assay showing that 231_control cells infiltrate the wounded area as single cells. Click here for file

Additional file 2 **Wound healing assay 231-E-cadherin cells**. Wound healing assay showing that 231_E-cadh cells invade the wounded area as cell clusters in a collective fashion. Click here for file

Additional file 3 **Wound healing assay 231-P-cadherin cells**. Wound healing assay showing that 231_P-cadh cells invade the wounded area as cell clusters in a collective fashion. Click here for file

Additional file 4 **Complete list of genes modulated by E-cadherin and/or P-cadherin (at least 2 fold with respect to control cells) in 231 cells**. Genes modulated by E-cadherin and/or P-cadherin are grouped by function and arranged by alphabetic order. The genes differentially expressed with statistically significance (FDR < 0.15) between E-cadherin and P-cadherin are highlighted in red bold letter. Click here for file

Additional file 5 **Potential signaling networks mediated by E-cadherin-regulated genes at the biological process level**. Genes modulated by E-cadherin may be involved in crosstalk among diverse biological processes. Green: down-regulated genes; Red: up-regulated genes. Semi-transparent nodes were inferred by the relevance networks algorithm due to their statistical relevance according to the user-defined network components. Click here for file

Additional file 6 **Potential signaling networks mediated by P-cadherin-regulated genes at the biological process level**. Genes modulated by P-cadherin may be involved in crosstalk among diverse biological processes. Green: down-regulated genes; Red: up-regulated genes. Click here for file
