# Supplementary material for: Functional characterization of E- and P-cadherin in invasive breast cancer cells
Source: BMC Cancer. 2009 Mar 3;9:74. doi: 10.1186/1471-2407-9-74 (PMC2656544; doi:10.1186/1471-2407-9-74)
Supplement: Additional file 4 — Complete list of genes modulated by E-cadherin and/or P-cadherin (at least 2 fold with respect to control cells) in 231 cells. Genes modulated by E-cadherin and/or P-cadherin are grouped by function and arranged by alphabetic order. The genes differentially expressed with statistically significance (FDR < 0.15) between E-cadherin and P-cadherin are highlighted in red bold letter. [file 1471-2407-9-74-S4.doc]

**Additional file 4. Complete list of genes modulated by E-cadherin and/or P-cadherin (at least 2 fold with respect to control cells) in 231 cells.** Genes are grouped by function and arranged by alphabetic order. The genes differentially expressed with statistically significance (FDR<0.15) between E-cadherin and P-cadherin are highlighted in red bold letter. Mean E-cadh: mean gene expression value in two E-cadherin clones; Mean P-cadh: mean gene expression value in two P-cadherin clones. Negative value indicates that gene expression is down-regulated with respect to control cells.

| **GenBank accession #** | **Gene Symbol** | **Description** | **Mean**  **E-cadh** | **Mean P-cadh** |
| --- | --- | --- | --- | --- |
| **Cell Adhesion & Extracellular Matrix (n=12)** | | | | |
| AA598561 | *CD164* | CD164 antigen, sialomucin | **2.62** | 1.89 |
| **AI671174** | *CDH1* | Cadherin 1, type 1, E-cadherin (epithelial) | **14.32** | 1.58 |
| **AA865745** | *CDH18* | Cadherin 18, type 2 | **2.07** | 1.09 |
| **AA425556** | *CDH3* | Cadherin 3, type 1, P-cadherin (placental) | 1.11 | **3.78** |
| **AI003692** | *CDH4* | Cadherin 4, type 1, R-cadherin (retinal) | **2.03** | -1.20 |
| H11732 | *CLECSF2* | C-type lectin domain family 2, member B | **4.45** | **3.86** |
| AA478481 | *COL12A1* | Collagen, type XII, alpha 1 | **2.90** | **2.01** |
| AW467461 | *ITGB2* | Integrin, beta 2 | **-2.28** | -1.98 |
| **N33214** | *MMP14* | Matrix metalloproteinase 14 (membrane-inserted) | -1.24 | **-2.46** |
| AA167273 | *SDC3* | Syndecan 3 (N-syndecan) | **2.60** | 1.52 |
| **N50444** | *SEMA4D* | semaphorin 4D | **2.20** | 1.30 |
| **N59721** | *SERPINE2* | Serine (or cysteine) proteinase inhibitor, clade E (plasminogen activator inhibitor type 1), member 2 | -1.38 | **-2.87** |
| **Motility & cytoskeleton (n=9)** | | | | |
| CR936693 | *ADD1* | Adducin 1 (alpha) | **-3.93** | **-4.66** |
| N49912 | *ARHGDIB* | Rho GDP dissociation inhibitor (GDI) beta | **-2.41** | **-6.07** |
| AA009697 | *CDC42* | Cell division cycle 42 (GTP binding protein, 25kDa) | **2.03** | 1.73 |
| AA148200 | *ILK* | Integrin-linked kinase-2 | **2.09** | 1.66 |
| AI348818 | *KLHL2* | Kelch-like 2, Mayven (Drosophila) | -1.27 | **-2.07** |
| AA074079 | *KRTAP4-7* | Keratin associated protein 4-7 | **-2.14** | -1.53 |
| AA968896 | *MDK* | Midkine (neurite growth-promoting factor 2) | **-2.53** | **-2.51** |
| T61428 | *NEDD9* | Neural precursor cell expressed, developmentally down-regulated 9 | 1.21 | **3.48** |
| R55105 | *SGCB* | Sarcoglycan, beta | **2.32** | 1.54 |
| **Signal Transduction (n=29)** | | | | |
| AA700054 | *ADFP* | Adipose differentiation-related protein | **-2.86** | -1.59 |
| BE747838 | *AKAP12* | A kinase (PRKA) anchor protein (gravin) 12 | **2.55** | **2.10** |
| **BM707300** | *CALR* | Calreticulin | -1.29 | **-2.01** |
| R19889 | *DAZAP2* | DAZ associated protein 2 | **-2.19** | **-2.16** |
| **AI140863** | *EDN2* | Endothelin 2 | **3.12** | 1.10 |
| **AA446994** | *FGFR4* | Fibroblast growth factor receptor 4 | -1.17 | **-2.06** |
| AA448277 | *FOXO1A* | Forkhead box O1A (rhabdomyosarcoma) | **3.10** | **2.47** |
| **H63934** | *GABRE* | Gamma-aminobutyric acid (GABA) A receptor, epsilon | 1.37 | **2.78** |
| **N70841** | *GABBR1* | Gamma-aminobutyric acid (GABA) B receptor, 1 | **2.14** | 1.25 |
| N20338 | *HGS* | Hepatocyte growth factor-regulated tyrosine kinase substrate | **2.16** | 1.53 |
| BE259102 | *HSPA2* | Heat shock 70kDa protein 2 | **-2.20** | -1.52 |
| AI075335 | *HSPA5* | Heat shock 70kDa protein 5 | -1.54 | **-3.18** |
| BE221241 | *IFI44* | Interferon-induced protein 44 | **-2.68** | -1.89 |
| **AA026831** | *KDR* | Kinase insert domain receptor (a type III receptor tyrosine kinase) | **3.33** | 1.09 |
| **H27986** | *LMO4* | LIM domain only 4 | **2.56** | 1.06 |
| AA455056 | *MAPKAPK2* | Mitogen-activated protein kinase-activated protein kinase 2 | **2.12** | 1.56 |
| **GenBank accession #** | **Gene Symbol** | **Description** | **Mean**  **E-cadh** | **Mean P-cadh** |
| **Signal Transduction (continuation)** | | | | |
|  |  |  |  |  |
| BM676842 | *MERTK* | C-mer proto-oncogene tyrosine kinase | **-2.28** | **-2.53** |
| W55872 | *NFKBIA* | Nuclear factor of kappa light polypeptide gene enhancer in B-cells inhibitor, alpha | **2.25** | 1.66 |
| W47106 | *NID67* | Putative small membrane protein NID67 | **2.25** | 1.59 |
| AA701502 | *PDGFA* | Platelet-derived growth factor alpha polypeptide | -1.14 | **-2.17** |
| AA478066 | *PKMYT1* | Protein kinase, membrane associated tyrosine/threonine 1 | 1.79 | **2.13** |
| **H26426** | *PTPRM* | Protein tyrosine phosphatase, receptor type, M | 1.23 | **-2.05** |
| AA156461 | *PTTG1IP* | Pituitary tumor-transforming 1 interacting protein | -1.84 | **-2.38** |
| **BM668271** | *RALBP1* | RalA binding protein 1 | **2.01** | 1.13 |
| **BM669560** | *SGK* | Serum/glucocorticoid regulated kinase | **2.33** | 1.39 |
| AA664389 | *TGFB1I4* | TSC22 domain family, member 1 | **-2.30** | **-2.31** |
| **AI268473** | *TLE1* | Transducin-like enhancer of split 1 | **2.00** | 1.15 |
| R71725 | *TRAF1* | TNF receptor-associated factor 1 | **2.11** | **2.26** |
| H07991 | *VEGFC* | Vascular endothelial growth factor C | **2.20** | **2.61** |
|  |  |  |  |  |
| **Transcription (n=12)** | | | | |
| H21041 | *ATF3* | Activating transcription factor 3 | **2.31** | 1.65 |
| **AA026120** | *BHLHB2* | Basic helix-loop-helix domain containing, class B, 2 | 1.04 | **-2.11** |
| **AA115076** | *CITED2* | Cbp/p300-interacting transactivator, with Glu/Asp-rich carboxy-terminal domain, 2 | 1.33 | **2.03** |
| AI040640 | *H2AFY* | H2A histone family, member Y | **2.43** | 1.10 |
| AI218900 | *HIST1H4B* | Histone 1, H4b | **2.08** | **2.14** |
| AA868008 | *HIST1H4C* | Histone 1, H4c | **2.50** | **2.15** |
| AI653010 | *HIST1H4J* | Histone 1, H4j | **2.02** | 1.99 |
| **AA496576** | *NFE2L1* | Nuclear factor (erythroid-derived 2)-like 1 | -1.32 | **-2.09** |
| AA456289 | *NFIA* | Nuclear factor I/A | 1.82 | **2.10** |
| R51836 | *PAX8* | Paired box gene 8 | **2.01** | 1.57 |
| **AA026102** | *TCF3* | Transcription factor 3 (E2A immunoglobulin enhancer binding factors E12/E47) | -1.56 | **-2.43** |
| AA016980 | *ZMAT1* | Zinc finger, matrin type 1 | -1.80 | **-2.92** |
|  |  |  |  |  |
| **Cell Cycle and apoptosis (n=18)** | | | | |
| AA459213 | *CCNA2* | Cyclin A2 | **2.86** | **2.32** |
| AA083032 | *CCNG1* | Cyclin G1 | **-2.24** | **-2.77** |
| **AA489752** | *CCNG2* | Cyclin G2 | **2.18** | -1.73 |
| R07261 | *CDCA8* | Cell division cycle associated 8 | **3.34** | 1.88 |
| R78607 | *CDK2AP1* | CDK2-associated protein 1 | **2.14** | 1.52 |
| R77517 | *CDKN2D* | Cyclin-dependent kinase inhibitor 2D (p19, inhibits CDK4) | **2.21** | 1.49 |
| AI369629 | *CENPA* | Centromere protein A, 17kDa | **2.19** | 1.77 |
| AA486628 | *EGR1* | Early growth response 1 | **4.20** | 1.53 |
| AA064616 | *FBXO5* | F-box protein 5 | **2.16** | **2.86** |
| AA292054 | *GAS1* | Growth arrest-specific 1 | **-2.18** | -1.58 |
| BM681758 | *GAS5* | Growth arrest-specific 5 | **-2.62** | -1.91 |
| **N94468** | *JUNB* | Homo sapiens jun B proto-oncogene (JUNB) | **2.05** | 1.04 |
| N93661 | *PCTK1* | PCTAIRE protein kinase 1 | **2.10** | 1.42 |
| **W01536** | *PDCD4* | Programmed cell death 4 (neoplastic transformation inhibitor) | -1.00 | **-2.32** |
| AA973492 | *PFTK1* | PFTAIRE protein kinase 1 | **2.45** | 1.65 |
| AA458838 | *PMAIP1* | phorbol-12-myristate-13-acetate-induced protein 1 | **2.02** | 1.76 |
| AA826373 | *RRM2* | Ribonucleotide reductase M2 polypeptide | **3.30** | **5.53** |
| AA039640 | *WEE1* | WEE1 homolog (S. pombe) | **4.78** | **2.24** |
|  |  |  |  |  |
| **GenBank accession #** | **Gene Symbol** | **Description** | **Mean**  **E-cadh** | **Mean P-cadh** |
| **Cytokines & Immune response (n=9)** | | | | |
|  |  |  |  |  |
| W46900 | *CXCL1* | Chemokine (C-X-C motif) ligand 1 (melanoma growth stimulating activity, alpha) | **3.56** | **2.58** |
| BF030509 | *FPR1* | Formyl peptide receptor 1 | **2.10** | 1.16 |
| **N31850** | *IL24* | Interleukin 24 | **2.88** | **-2.16** |
| AA464595 | *KISS1* | KiSS-1 metastasis-suppressor | **-2.15** | -1.89 |
| **W73144** | *LCP1* | Lymphocyte cytosolic protein 1 (L-plastin) | -1.06 | **-2.36** |
| N67017 | *LIFR* | Leukemia inhibitory factor receptor | **2.30** | 1.90 |
| AA664219 | *NR3C1* | Nuclear receptor subfamily 3, group C, member 1 (glucocorticoid receptor) | **2.12** | 1.38 |
| **AA485355** | *SOCS1* | Suppressor of cytokine signaling 1 | **2.43** | 1.04 |
| R70479 | *TNFAIP3* | Tumor necrosis factor, alpha-induced protein 3 | **2.11** | 1.24 |
|  |  |  |  |  |
| **Basic Cellular Functions (n=34)** | | | | |
| **AA429895** | *ABCC3* | ATP-binding cassette, sub-family C (CFTR/MRP), member 3 | -1.76 | **-3.23** |
| **H25923** | *ABCD3* | ATP-binding cassette, sub-family D (ALD), member 3 | 1.01 | **2.03** |
| BE541448 | *ALG6* | Asparagine-linked glycosylation 6 homolog (yeast, alpha-1,3-glucosyltransferase) | **-2.50** | -1.42 |
| **AA598814** | *ATP1B1* | ATPase, Na+/K+ transporting, beta 1 polypeptide | **2.99** | 1.67 |
| AA281733 | *EIF1AX* | Eukaryotic translation initiation factor 1A, X-linked | **2.57** | **3.49** |
| BE396283 | *EIF3S1* | Eukaryotic translation initiation factor 3, subunit 1 | **-2.02** | -1.39 |
| H56918 | *EIF4A1* | Eukaryotic translation initiation factor 4A, isoform 1 | **-2.36** | -1.39 |
| H94332 | *FANCC* | Fanconi anemia, complementation group C | -1.76 | **-2.59** |
| N28486 | *FBXO32* | F-box protein 32 | **-2.06** | **-2.25** |
| AA015892 | *MARS* | Methionine-tRNA synthetase | **-2.07** | -1.99 |
| AA448637 | *MRRF* | Mitochondrial ribosome recycling factor | **-2.08** | -1.42 |
| AI287777 | *MSH6* | Homo sapiens mutS homolog 6 (E. coli) (MSH6), mRNA. | **2.06** | **2.02** |
| BE546134 | *NCBP2* | Nuclear cap binding protein subunit 2, 20kDa | -1.36 | **-2.25** |
| AA486626 | *PABPC1* | Poly(A) binding protein, cytoplasmic 1 | **2.06** | 1.34 |
| BE567355 | *PIN4* | Protein (peptidyl-prolyl cis/trans isomerase) NIMA-interacting, 4 (parvulin) | **2.79** | **3.03** |
| N72215 | *PSAP* | Prosaposin (variant Gaucher disease and variant metachromatic leukodystrophy) | -1.83 | **-2.03** |
| AA416733 | *PSMB2* | Proteasome (prosome, macropain) subunit, beta type, 2 | 1.63 | **2.10** |
| **AA282599** | *RNU22* | RNA, U22 small nucleolar | **-2.11** | 1.07 |
| BF305859 | *ROR1* | Receptor tyrosine kinase-like orphan receptor 1 | **-2.06** | -1.60 |
| AA662084 | *RPL37* | Ribosomal protein L37 | **-2.27** | **-2.07** |
| AA668301 | *RPS16* | Ribosomal protein S16 | **-2.14** | -1.63 |
| T74714 | *RPS6KA2* | Ribosomal protein S6 kinase, 90kDa, polypeptide 2 | 1.31 | **2.25** |
| R22239 | *SKP2* | S-phase kinase-associated protein 2 (p45) | 1.43 | **2.19** |
| AA453823 | *SLC1A3* | Solute carrier family 1 (glial high affinity glutamate transporter), member 3 | **-2.38** | **-2.23** |
| **AA406551** | *SLC2A3* | Solute carrier family 2 (facilitated glucose transporter), member 3 | 1.52 | **-2.43** |
| AA017382 | *SND1* | Staphylococcal nuclease domain containing 1 | **2.34** | **2.53** |
| BE249912 | *SPINT1* | Serine protease inhibitor, Kunitz type 1 | **-2.51** | -1.98 |
| **BE250007** | *SPINT2* | Serine protease inhibitor, Kunitz type, 2 | -1.14 | **-2.39** |
| BE264393 | *SQSTM1* | Sequestosome 1 | **-3.54** | **-3.30** |
| N30412 | *SRP46* | Splicing factor, arginine/serine-rich, 46kD | -1.77 | **-2.13** |
| **GenBank accession #** | **Gene Symbol** | **Description** | **Mean**  **E-cadh** | **Mean P-cadh** |
| **Basic Cellular Functions (continuation)** | | | | |
| N57744 | *TPT1* | Tumor protein, translationally-controlled 1 | -1.85 | **-2.06** |
| AA446748 | *TST* | Thiosulfate sulfurtransferase (rhodanese) | **2.25** | 1.27 |
| BE207600 | *UBC* | Ubiquitin C | **-2.12** | -1.42 |
| W90128 | *XBP1* | X-box binding protein 1 | **-2.13** | **-2.32** |
|  |  |  |  |  |
| **Metabolism (n=11)** | | | | |
| T98355 | *ELOVL6* | ELOVL family member 6, elongation of long chain fatty acids (FEN1/Elo2, SUR4/Elo3-like, yeast) | 1.53 | **2.46** |
| BE566343 | *GLRX* | Homo sapiens glutaredoxin (thioltransferase), mRNA | **-2.83** | **-2.59** |
| AA035347 | *GNS* | Glucosamine (N-acetyl)-6-sulfatase (Sanfilippo disease IIID) | **-2.70** | -1.80 |
| BU731927 | *HMOX1* | Heme oxygenase (decycling) 1 | **-2.46** | **-2.52** |
| **BE548504** | *KYNU* | Kynureninase (L-kynurenine hydrolase) | -1.53 | **-2.16** |
| AI357590 | *OAS3* | 2'-5'oligoadenylate synthetase 3 (OAS3) mRNA | **2.21** | 1.43 |
| AW247294 | *PGD* | Phosphogluconate dehydrogenase | **-2.28** | -1.65 |
| AA151486 | *PRPS2* | Phosphoribosyl pyrophosphate synthetase 2 | **2.54** | **2.02** |
| AI015679 | *PSAT1* | Phosphoserine aminotransferase 1 | **-2.02** | **-2.03** |
| R00707 | *SCD* | Stearoyl-CoA desaturase (delta-9-desaturase) | **-2.49** | -1.51 |
| AA620477 | *SHMT2* | Serine hydroxymethyltransferase 2 (mitochondrial) | **-2.28** | -1.79 |
|  |  |  |  |  |
| **Unknown Function (n=16)** | | | | |
| **H80103** | *C18orf55* | Chromosome 18 open reading frame 55 | 1.14 | **2.19** |
| AA608713 | *C1QDC1* | C1q domain containing 1 | **-2.34** | -1.74 |
| H80685 | *C5orf13* | Chromosome 5 open reading frame 13 | **2.04** | **2.09** |
| W90323 | *C6orf173* | Chromosome 6 open reading frame 173 | 1.61 | **2.26** |
| AA453289 | *C9orf60* | Zyg-11 homolog B (C. elegans)-like | **-2.35** | **-2.00** |
| R08270 | *CXorf6* | Chromosome X open reading frame 6 | **2.44** | 1.94 |
| AI262933 | *EIF1AP1* | eukaryotic translation initiation factor 1A pseudogene 1 (EIF1AP1) on chromosome 1 | **2.26** | **2.39** |
| N94612 | *FLJ11305* | Hypothetical protein FLJ11305 | **-2.30** | -1.94 |
| W95041 | *HS3ST3B1* | Hypothetical protein MGC12916 | 1.90 | **2.52** |
| W87747 | *LOC399715* | FLJ46311 protein | **2.14** | **2.18** |
| R20669 | *LRRC28* | Leucine rich repeat containing 28 | -1.73 | **-2.24** |
| AI017797 | *MFHAS1* | Malignant fibrous histiocytoma amplified sequence 1 | **2.15** | 1.96 |
| AA001918 | *MI-ER1* | Mesoderm induction early response 1 homolog (Xenopus laevis) | **2.03** | 1.49 |
| AA431741 | *MLF1IP* | MLF1 interacting protein | 1.85 | **2.02** |
| AA251026 | *TMEM32* | Transmembrane protein 32 | 1.62 | **2.16** |
| AA060698 | *R23877* | Transcribed locus | -1.25 | **-2.35** |
|  |  |  |  |  |
