# Supplementary material for: Functional characterization of E- and P-cadherin in invasive breast cancer cells
Source: BMC Cancer. 2009 Mar 3;9:74. doi: 10.1186/1471-2407-9-74 (PMC2656544; doi:10.1186/1471-2407-9-74)
Supplement: Additional file 5 — Potential signaling networks mediated by E-cadherin-regulated genes at the biological process level. Genes modulated by E-cadherin may be involved in crosstalk among diverse biological processes. Green: down-regulated genes; Red: up-regulated genes. Semi-transparent nodes were inferred by the relevance networks algorithm due to their statistical relevance according to the user-defined network components. [file 1471-2407-9-74-S5.pdf]

GENES MODULATED BY E-CADHERIN MAY BE INVOLVED IN CROSSTALK AMONG DIVERSE BIOLOGICAL PROCESSES

Down-regulated by E-cadherin

Up-regulated by E-cadherin

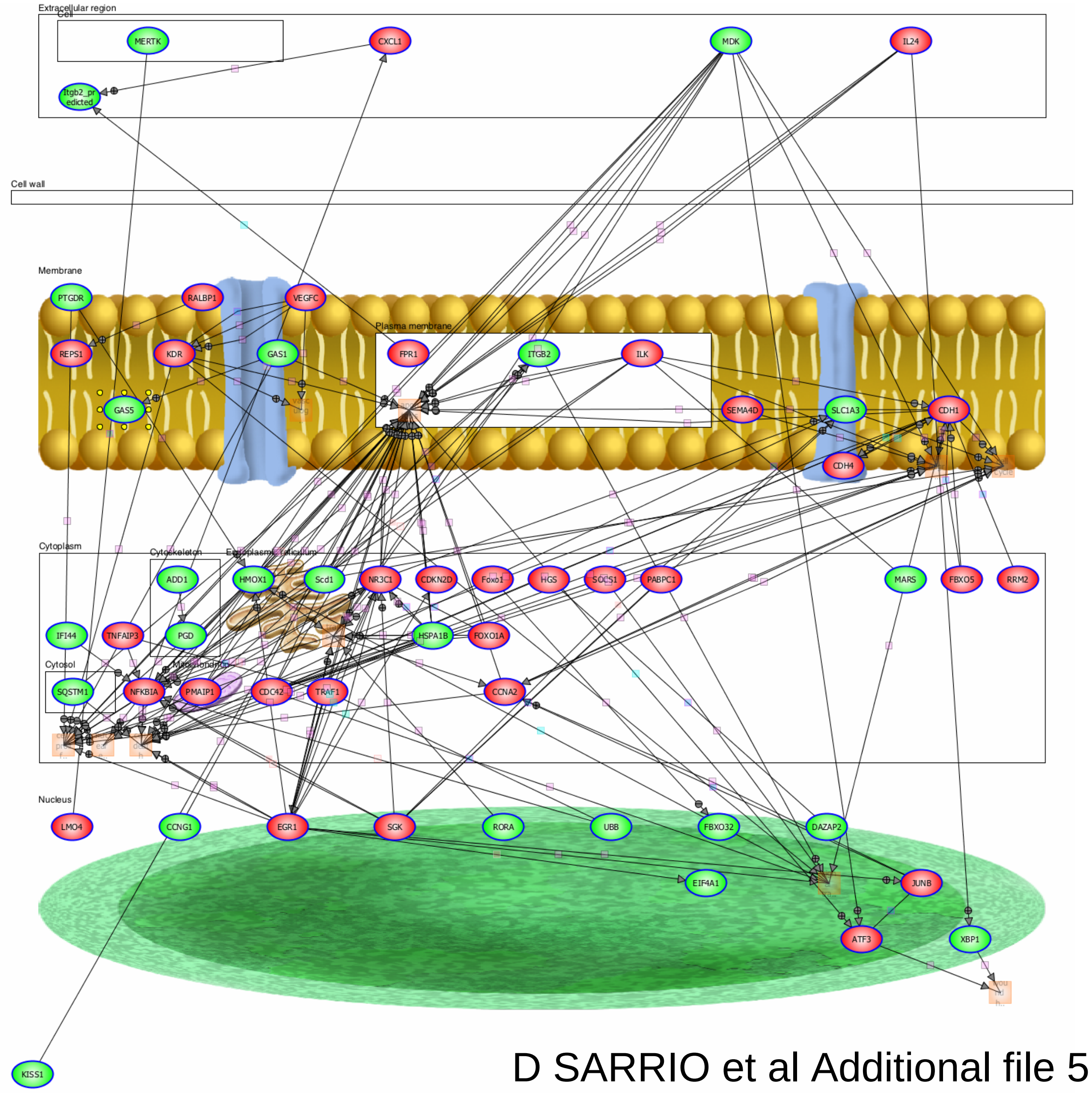

D SARRIO et al Additional file 5
