# Supplementary material for: Functional characterization of E- and P-cadherin in invasive breast cancer cells
Source: BMC Cancer. 2009 Mar 3;9:74. doi: 10.1186/1471-2407-9-74 (PMC2656544; doi:10.1186/1471-2407-9-74)
Supplement: Additional file 6 — Potential signaling networks mediated by P-cadherin-regulated genes at the biological process level. Genes modulated by P-cadherin may be involved in crosstalk among diverse biological processes. Green: down-regulated genes; Red: up-regulated genes. [file 1471-2407-9-74-S6.pdf]

# GENES MODULATED BY P-CADHERIN MAY BE INVOLVED IN CROSSTALK AMONG DIVERSE BIOLOGICAL PROCESSES

Down-regulated by P-cadherin

Up-regulated by P-cadherin

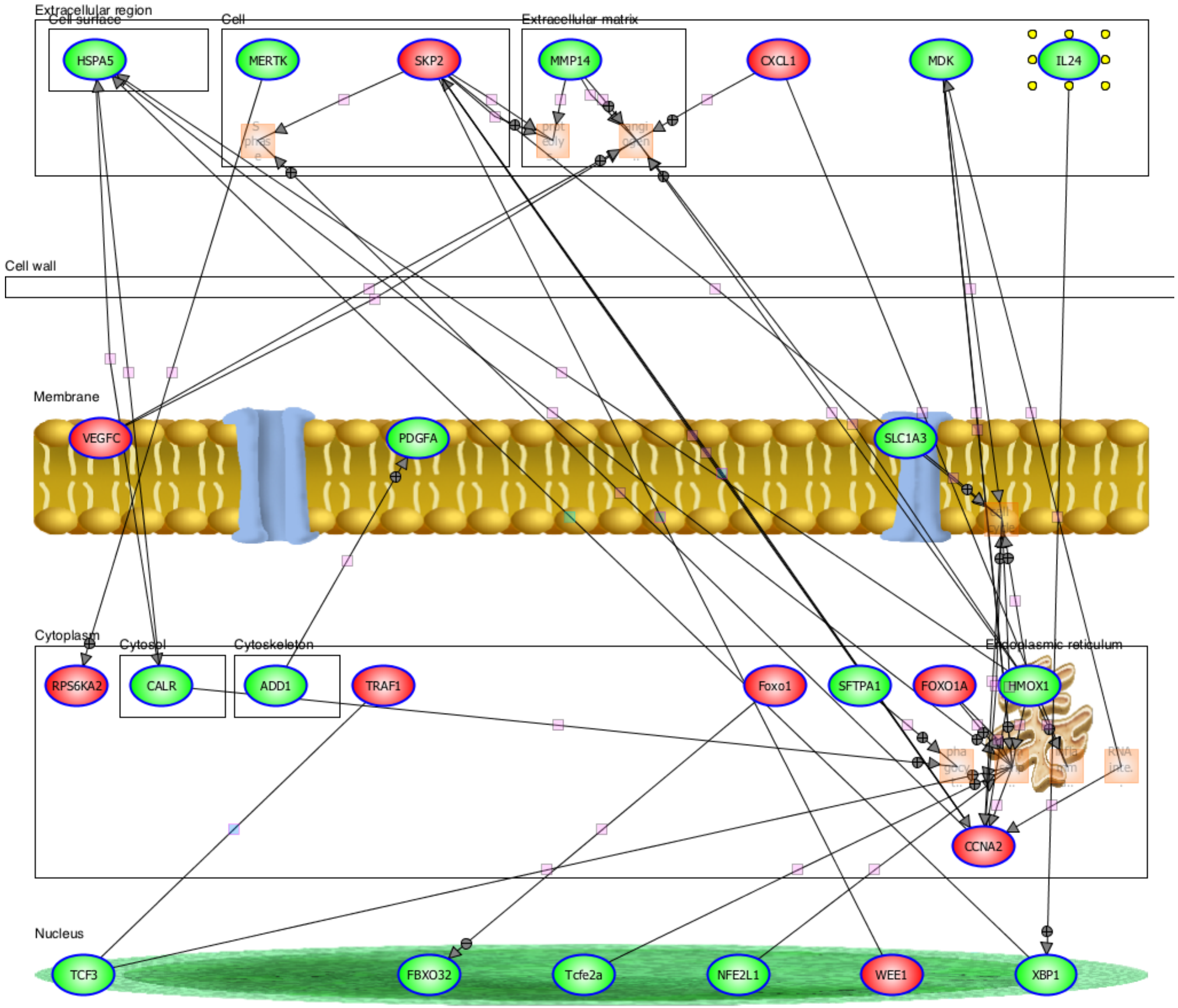


D SARRIO

Additional file 6
